# PEG-lipid micelles enable cholesterol efflux in Niemann-Pick Type C1 disease-based lysosomal storage disorder

**DOI:** 10.1038/srep31750

**Published:** 2016-08-30

**Authors:** Anna Brown, Siddharth Patel, Carl Ward, Anna Lorenz, Mauren Ortiz, Allison DuRoss, Fabian Wieghardt, Amanda Esch, Elsje G. Otten, Laura M. Heiser, Viktor I. Korolchuk, Conroy Sun, Sovan Sarkar, Gaurav Sahay

**Affiliations:** 1 Department of Pharmaceutical Sciences, College of Pharmacy, Collaborative Life Science Building, Oregon State University, Portland OR, USA; 2 Institute of Cancer and Genomic Sciences, Institute of Biomedical Research, College of Medical and Dental Sciences, University of Birmingham, Edgbaston, Birmingham B15 2TT, United Kingdom; 3 Department of Biomedical Engineering, Collaborative Life Science Building, Oregon Health Science University, Portland OR, USA; 4 Institute for Cell and Molecular Biosciences, Newcastle University Institute for Ageing, Campus for Ageing and Vitality, Newcastle University, Newcastle upon Tyne NE4 5PL, United Kingdom; 5 Department of Radiation Medicine, School of Medicine, Oregon Health Science University, Portland OR, USA

## Abstract

2-Hydroxy-propyl-β-cyclodextrin (HPβCD), a cholesterol scavenger, is currently undergoing Phase 2b/3 clinical trial for treatment of Niemann Pick Type C-1 (NPC1), a fatal neurodegenerative disorder that stems from abnormal cholesterol accumulation in the endo/lysosomes. Unfortunately, the extremely high doses of HPβCD required to prevent progressive neurodegeneration exacerbates ototoxicity, pulmonary toxicity and autophagy-based cellular defects. We present unexpected evidence that a poly (ethylene glycol) (PEG)-lipid conjugate enables cholesterol clearance from endo/lysosomes of *Npc1* mutant (*Npc1*^−/−^) cells. Herein, we show that distearyl-phosphatidylethanolamine-PEG (DSPE-PEG), which forms 12-nm micelles above the critical micelle concentration, accumulates heavily inside cholesterol-rich late endosomes in *Npc1*^−/−^ cells. This potentially results in cholesterol solubilization and leakage from lysosomes. High-throughput screening revealed that DSPE-PEG, in combination with HPβCD, acts synergistically to efflux cholesterol without significantly aggravating autophagy defects. These well-known excipients can be used as admixtures to treat NPC1 disorder. Increasing PEG chain lengths from 350 Da-30 kDa in DSPE-PEG micelles, or increasing DSPE-PEG content in an array of liposomes packaged with HPβCD, improved cholesterol egress, while Pluronic block copolymers capable of micelle formation showed slight effects at high concentrations. We postulate that PEG-lipid based nanocarriers can serve as bioactive drug delivery systems for effective treatment of lysosomal storage disorders.

Niemann Pick Type C (NPC) is an inherited autosomal recessive lysosomal storage disorder that affects approximately 1:120,000 children globally and results in hepatomegaly, progressive neurodegeneration, and finally death^1^,^2^. There are no current treatments available for children suffering from this devastating rare genetic disease. 95% of NPC cases result from defects in the NPC1 protein, a large glycoprotein with 13-membrane-spanning domains that resides on the surface of late endosomes/lysosomes, with a sterol-sensing domain in the lumen that binds to cholesterol and enables egress to a cytoplasmic location^3^. NPC1 deficiency causes cholesterol accumulation that manifests as enlargement of late endosomes/lysosomes, ultimately leading to neurodegeneration, especially loss of Purkinje fibers in the cerebellum, which leads to ataxia and cognitive loss^4^,^5^. HPβCD is a Food and Drug Administration (FDA) approved hydrophilic small molecule excipient that has a hydrophobic pocket capable of binding and solubilizing cholesterol^6^. It is capable of the efflux and redistribution of cholesterol in mammalian cells^7^; however, it does not readily cross the blood brain barrier (only 0.2% reaches the brain on systemic administration). In preclinical studies, a direct brain injection of HPβCD into an NPC mouse model slowed the progression of neuronal loss and improved its survival^8^,^9^,^10^,^11^. Similar results were observed in a feline model of NPC disease, with an intra-cisternal administration used to decrease neurodegeneration and prolong survival^12^. More recently, an intrathecal cyclodextrin injection in a single NPC1 patient led to an increase in cholesterol redistribution in the central nervous system and improved vertical gaze palsy, a clinical indicator of NPC-linked neurodegeneration^13^. Based on these results, HPβCD has entered NIH initiated Phase 2b/3 clinical trial, and the FDA has granted orphan drug status to further accelerate use of HPβCD for treating NPC disease. An impediment to the clinical translation of HPβCD is the exorbitant amount of drug required to clear cholesterol in order to elicit a significant response. In a single example, a biweekly administered dose of 200 mg, with a total of 27 injections, applied by lumbar puncture to a child with NPC, slowed neurodegeneration but resulted in high-frequency hearing loss^13^. In the feline model, a subcutaneous injection of 8000 mg/kg was necessary to halt the loss of Purkinje neurons, but resulted in significant pulmonary toxicity^12^.

Liposomes are among the most advanced drug delivery systems for effective intracellular delivery, and thus an obvious carrier choice for delivery of HPβCD to remove intracellular cholesterol aggregates^14^,^15^,^16^. Liposomal drug delivery vectors have several advantages, including prolonged circulation times, the potential to incorporate multiple drugs with different mechanisms of action and solubility, and surface decoration with a variety of targeting ligands (e.g. transferrin) that can enable transport across the blood brain barrier^14^,^17^,^18^. Lipid nanoparticles (LNPs), a subset of liposomal vectors, have been very effective in the delivery of nucleic acids^19^,^20^. LNPs are shown to enter cells through endocytic portals and are selectively recycled through NPC1-positive late endosomes; the absence of NPC1 showed a 10–15-fold retention of LNPs inside cells which translated to improved nucleic acid delivery^21^. Hydrogenated soy phosphatidylcholine (HSPC) based liposomes that incorporate water-soluble small molecules in the core had a similar pattern of retention in the endo/lysosomal system of NPC1 deficient cells^21^. Based on these studies, we hypothesized that simple liposomal formulations would enhance site-specific delivery of HPβCD into the cholesterol rich endo/lysosomes in *Npc1* deficient mouse embryonic fibroblasts (MEFs). We created a small set of two-component liposomes from HSPC and distearyl-phosphatidylethanolamine conjugated with polyethylene glycol (DSPE-PEG), a common PEG-lipid known to improve nanocarrier stability. Surprisingly, we found that increasing the molar ratio of DSPE-PEG in the liposomes improved HPβCD mediated cholesterol efflux, and ultimately deduced that DSPE-PEG on its own triggered cholesterol clearance. Most remarkably, we found that DSPE-PEG used in combination with HPβCD showed a substantial decline in the lysosomal cholesterol content in *Npc1*-deficient cells. These studies strongly suggest that a simple mixture of two well-characterized biocompatible excipients can have a significant impact in the treatment of NPC lysosomal storage disorder.

## Results

**Figure 1:**
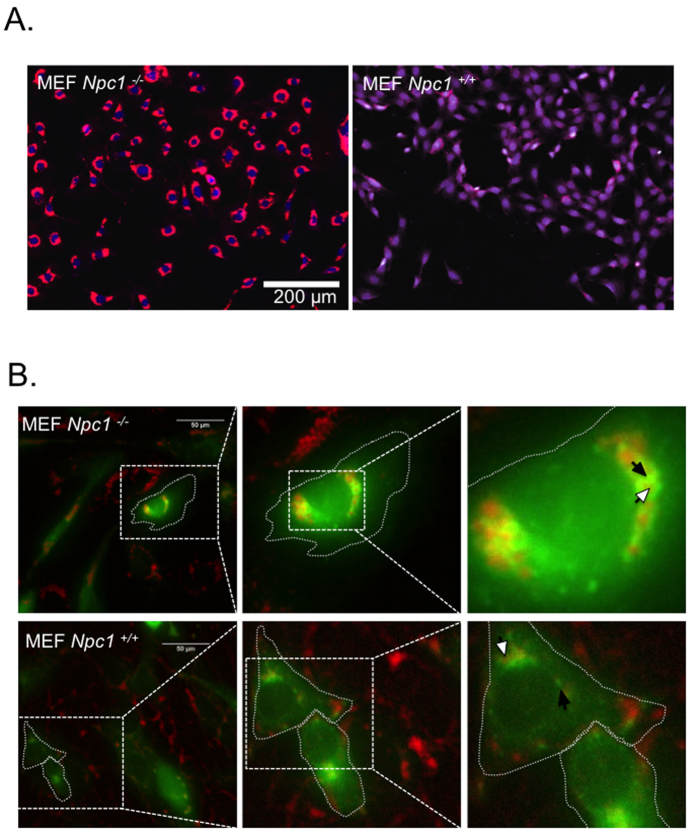
*Npc1* deficiency leads to cholesterol accumulation in enlarged late endosomes/lysosomes. (**A**) Filipin staining was performed to visualize subcellular cholesterol in *Npc1*^−/−^ and *Npc1*^+/+^ MEFs. A high throughput microscope was used to compare subcellular cholesterol (psuedo colored red) in both cell types. Nucleus was stained using HCS nuclear mask deep red stain. (psuedo colored blue) (**B**) *Npc1*^−/−^ and *Npc1*^+/+^ MEFs were transfected with to Rab-7 GFP (pseudo colored green) using a baculovirus. Cells were fixed 18 hours post transfection and stained with filipin (red). The cell outline is demarcated with dotted line; white arrow shows filipin and black arrow endosomal Rab7-GFP. All experiments were conducted in triplicates.

A filipin-based assay was used to visualize accumulation of cholesterol in *Npc1*^−/−^ MEFs. Filipin interacts with cholesterol to form a complex that is fluorescent at 405 nm. This assay has been utilized as a clinical diagnostic test for NPC disease and allows for rapid identification of molecules capable of sequestering cholesterol from intracellular membranes^22^,^23^. Using this assay, we confirmed heavy accumulation of cholesterol in enlarged punctate structures inside *Npc1*^−/−^ MEFs, relative to *Npc1*^+/+^ MEFs (Fig. 1A). To characterize these structures, we transfected both cell lines with Rab7-GFP, a late-endosome marker. Fluorescence microscopy revealed enlarged late-endosomes in *Npc1*^−/−^ cells, with a clear demarcation of Rab7-GFP on the lysosomal surface. Filipin-stained cholesterol was observed within the lumen of Rab7-positive late-endosomes (Fig. 1B). In contrast, *Npc1*^+/+^ cells showed smaller Rab7-positive punctate structures, and the filipin stain appeared substantially lower and markedly more diffused in the cytoplasm (Fig. 1B), suggestive of normal cholesterol transport in these cells. These studies confirm abnormal accumulation of cholesterol in late endo/lysosomes of *Npc1*^−/−^ MEF cells.

**Figure 2:**
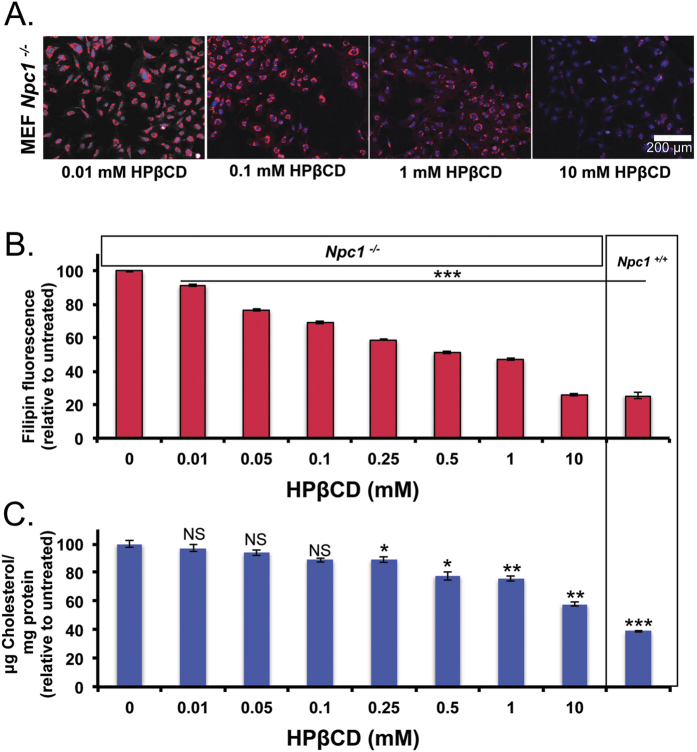
HPβCD removes cholesterol from *Npc1*^−/−^ cells. *Npc1*^−/−^ MEFs were incubated with HPβCD (0–10 mM) for 24 hours. Cells were either fixed or lysed. (**A**) Fixed cells were imaged for filipin stain using high throughput imaging and representative images are show for 0.01, 0.1, 1 and 10 mM HPβCD treatments. (**B**) Filipin fluorescence per cell was quantified using Olympus scanR analysis software, and normalized to untreated cells. 48 images per treatment group were analyzed. (**C**) Cell lysates were analyzed for total cellular cholesterol using the amplex red assay and normalized by total protein content. Untreated *Npc1*^+/+^ MEFs are shown for comparison. Results were tested for statistical significance using one-way ANOVA with Tukey’s multiple comparison test. *P < 0.05, **P < 0.01, ***P < 0.001 and NS = not significant.

We further utilized filipin imaging to demonstrate the well-documented use of HPβCD to decrease intracellular cholesterol from endo/lysosomes in *Npc1*^−/−^ cells, by cholesterol solubilization, which perhaps enables efflux from these intracellular depots. *Npc1*^−/−^ cells show a marked decrease in filipin fluorescence with increasing concentration of HPβCD treatment, up to 10 mM (Fig. 2A). Quantitative analysis of filipin staining shows that a 0.5 mM HPβCD treatment for 24 hours reduced endosomal cholesterol by 50%. A 10 mM HPβCD treatment further decreased cholesterol levels in *Npc1*^−/−^ cells (ca. 75–80%), which is comparable to the levels in *Npc1*^+/+^ MEFs (Fig. 2B). In addition, we used an amplex-red assay to quantify cholesterol in whole cell lysates. We confirm a dose-dependent decrease in the total cholesterol in *Npc1*^−/−^ cells (Fig. 2C).

We initially set out to encapsulate HPβCD in the hydrophilic core of liposomes, formulated from HSPC and DSPE-PEG_2k,_ in order to site-specifically deliver HPβCD into the endo/lysosomal system. DSPE-PEG_2k_ is a known liposomal stabilizer, and we tested liposomes composed of different molar ratios of these two lipids in order to optimize the formulation. Liposomes were assembled by thin film rehydration in the presence of a concentrated HPβCD solution and dialyzed to remove free cyclodextrin. Liposomes were characterized for their size, shape, and encapsulation efficiency. We confirmed liposomal assembly using Transmission Electron Microscopy (TEM) imaging and measured their average hydrodynamic sizes using Dynamic light scattering (DLS). Both DLS and TEM showed that increasing PEG-lipid content decreased the size of the nanoparticles from 150 nm to 10–15 nm (Fig. S1A,B). However, in formulations containing both lipid components, DLS suggested the presence of a mixed population of liposomes and micelles. We measured the loading efficiency using LC/MS/MS and found a gradual decrease in encapsulation with increasing PEG-lipid, which may be due to the increasing hydrophobic microenvironment of the core along with the decreasing size of the carrier. Surprisingly, when testing these formulations on *Npc1*^−/−^ cells, the nanocarriers with higher molar ratios DPSE-PEG_2k_ were the most potent in terms of cholesterol clearance despite a relatively lower loading efficiency of HPβCD (Fig. S1C).

**Figure 3:**
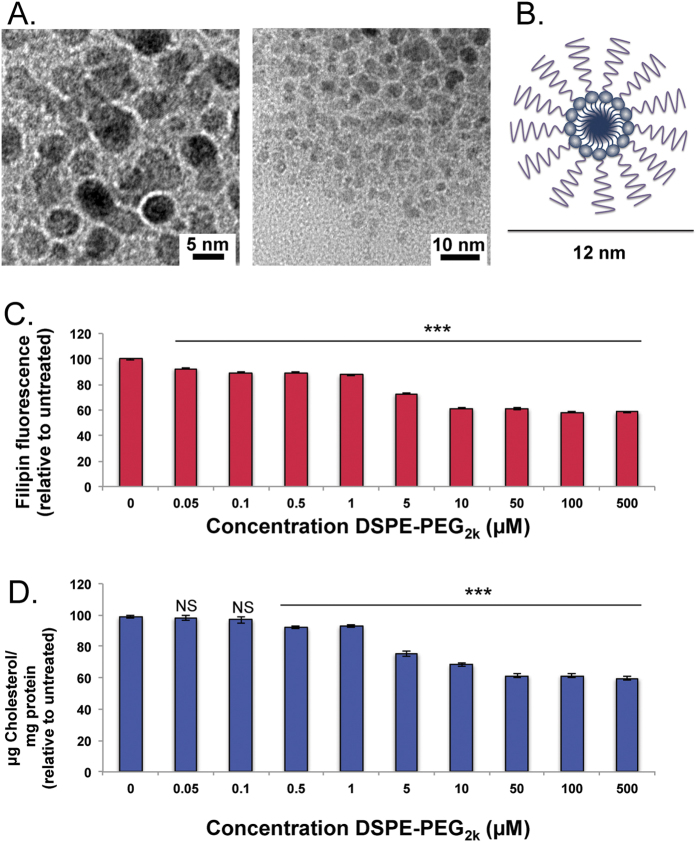
DSPE-PEG_2k_ reduces cellular cholesterol in *Npc1*^−/−^ cells with a dose dependent response. DSPE-PEG_2k_ micelles were assembled by thin film rehydration method. (**A**) TEM images of micelles show electron dense cores that are approximately 5 nm. (**B**) Schematic diagram of assembled DSPE-PEG_2k_ micelles has an electron dense hydrophobic core, with extended hydrophilic PEG head groups. (**C**) *Npc1*^−/−^ cells were exposed to different concentrations of DSPE-PEG_2k_ (0–500 μM), fixed and a dose dependent decrease in filipin fluorescence per cell was observed by high throuhgput screening. (**D**) Whole cell cholesterol (per mg protein), normalized to un-treated cells, also shows a dose dependent reduction in cholesterol when treated with DSPE-PEG_2k_. Results were tested for statistical significance using one-way ANOVA with Tukey’s multiple comparison test. *P < 0.05, **P < 0.01, ***P < 0.001 and NS = not significant.

DSPE-PEG_2k_ forms spherical micelles above the critical micelle concentration (CMC), of 1 μM. DLS of assembled micelles showed a consistent and uniform 12 nm hydrodynamic size and TEM imaging showed a 5 nm electron dense core (Fig. 3A). This data is consistent with published models of PEG-lipid micelles that self-assemble to form a core-shell structure, in which the DSPE tail aggregates into a hydrophobic core and PEG chains are exposed to the aqueous solvent (Fig. 3B). To our surprise, treatment with only DSPE-PEG_2k_ micelles resulted in a dose-dependent decrease in cholesterol levels in *Npc1*^−/−^ cells (Fig. 3C,D). Treatments as low as 1 μM DSPE-PEG_2k_ led to 13% reduction in cholesterol, which, when increased to 10 μM, resulted in a ~40% reduction in cholesterol. The 40% reduction was the highest cholesterol egress achieved, even with concentrations up to 500 μM. This decrease was significant, since a 75–80% reduction of cholesterol in *Npc1*^−/−^ cells reduced cholesterol to normal cellular levels (Fig. 2B,C). Both filipin and amplex red cholesterol assays show similar results, which strongly suggests that these materials are capable of reducing intracellular cholesterol deposits and enabling cholesterol efflux from the endo/lysosomal system.

**Figure 4:**
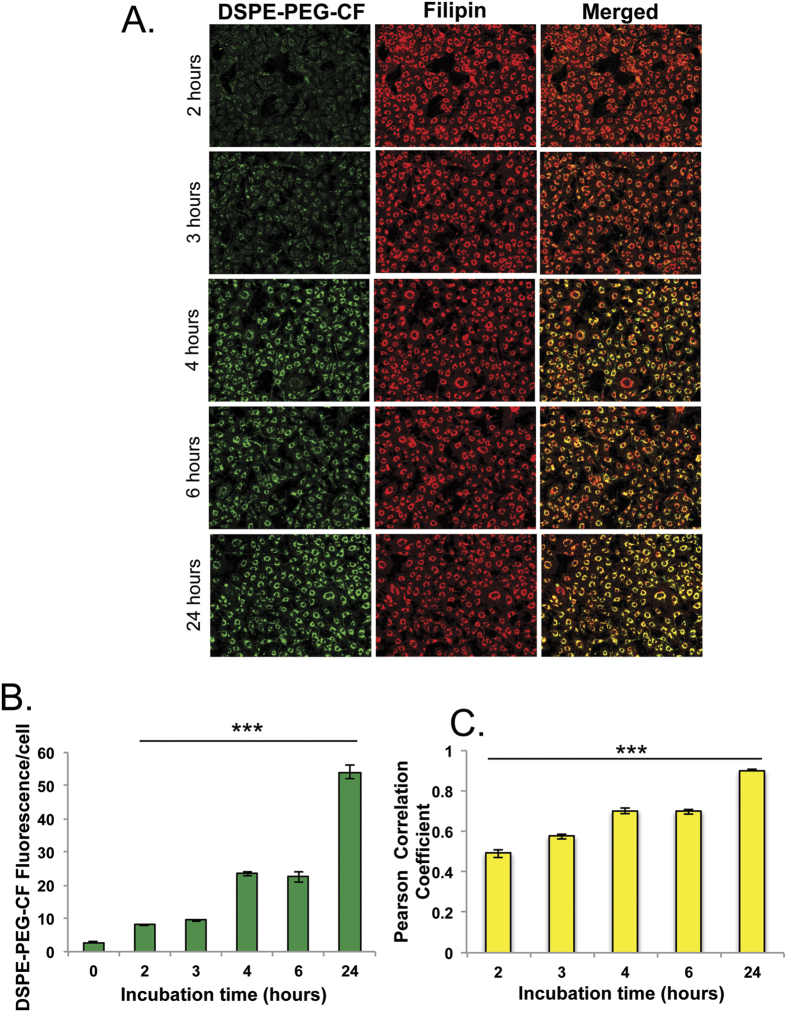
Labeled DSPE-PEG_2k_ is internalized and localized to filipin positive vesicles in *Npc1*^−/−^ cells. Carboxy-Fluorescein (CF) labeled DSPE-PEG_2k_ assembled into micelles by thin film rehydration was exposed to cells at 10 μM concentration for different time points (2, 3, 4, 6, 24 hours), cells were washed, fixed and filipin stained. (**A**) High throughput imaging (16 images/well) was performed to monitor DSPE-PEG_2k_-CF uptake and co-localization with endosomal cholesterol. (**B**) Image-J software was utilized to measure DSPE-PEG_2k_-CF uptake and (**C**) Monitor co-localization by calculation of Pearson Coefficient. (n = 5). Results were tested for statistical significance using one-way ANOVA with Tukey’s multiple comparison test relative to untreated cells (**B**) or 24 hour treated cells (**C**). *P < 0.05, **P < 0.01, ***P < 0.001 and NS = not significant.

DSPE-PEG_2k_ is an essential component of LNPs that have been shown to preferentially accumulate in the late endosomes/lysosomes of *Npc1*^−/−^ cells. Therefore, we hypothesized that DSPE-PEG_2k_ actively accumulates in these cells and subsequently leads to solubilization of cholesterol. To test this hypothesis, we evaluated the uptake kinetics of carboxy-fluorescein (CF) labeled DSPE-PEG_2k_ micelles at different time points and monitored their localization within filipin-positive late-endosomes using automated high-throughput imaging. The DSPE-PEG_2k_-CF was detected by cellular internalization after approximately 2 hours, and saturated the endo/lysosomal system within 24 hours; the relatively slow rate of uptake is suggestive of macropinocytosis (Fig. 4A,B). We utilized filipin to measure localization of DSPE-PEG_2k_-CF delivered to the cholesterol-rich late endosomes/lysosomes in *Npc1*^−/−^ cells. At 2 hours, the labeled micelles show ~50% co-localization with filipin-stained cholesterol deposits (Fig. 4C) but within 4–6 hours ~60% of the labeled micelles reached filipin-positive endosomes and by 24 hours we observed an 80% co-localization with late endosomes/lysosomes (Fig. 4C). These studies clearly show a substantial delivery of DSPE-PEG_2k_-CF micelles into the cholesterol-laden endo/lysosomes of *Npc1*^−/−^ cells. It is likely that DSPE-PEG interacts with large cholesterol deposits in the late endosomes, which leads to a degree of aggregate solubilization and potentially releases them from the lysosomal compartment. Interestingly, DSPE-PEG_2k_-CF did not show enhanced accumulation in wild-type cells (data not shown), suggestive of enhanced accumulation in late-endosomes due to recycling defects present in *Npc1*^−/−^ cells as previously observed^21^.

**Figure 5:**
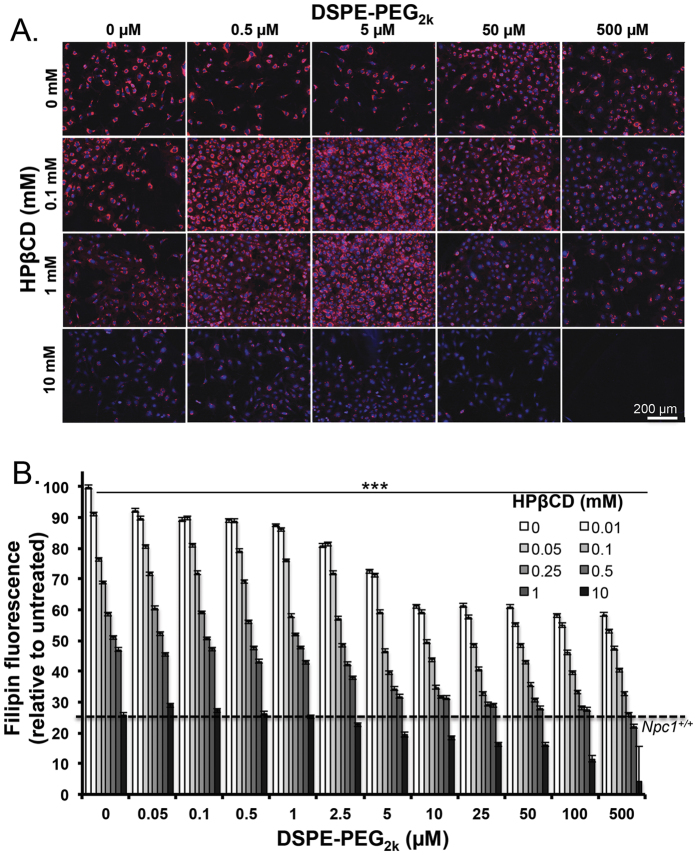
Combination treatment of PEG-lipid and HPBCD lead to synergistic clearance in *Npc1*^−/−^ cells. *Npc1*^−/−^ MEFs cells were treated with DSPE-PEG_2k_ micelles (0–500 μM) and HPβCD (0–10 mM). Cells were fixed and stained with filipin and nuclear mask. (**A**) Representative images of select treatments are shown and illustrate the synergistic cholesterol reduction of the two treatments. (**B**) Cells were quantitatively analyzed for filipin fluorescence per cell, and the dose dependent reduction of filipin fluorescence is shown normalized to untreated cells. Images per well were taken (n = 3, 16 images/well) and cells were quantified for total filipin fluorescence. The treatment with 500 μM DSPE-PEG_2k_ and 10 mM HPβCD is toxic, and no cells are observed. Results were tested for statistical significance using one-way ANOVA with Tukey’s multiple comparison test. *P < 0.05, **P < 0.01, ***P < 0.001 and NS = not significant.

We set out to evaluate the synergy of combinatorial treatments with both DSPE-PEG and HPβCD to potentiate cholesterol removal. We created a matrix of treatment groups consisting of DSPE-PEG_2k_ micelles (0.05–500 μM) and HPβCD ranging from 0.01 mM–10 mM. *Npc1*^−/−^ cells were exposed to these treatments, in combination or alone, for 24 hours, prior to fixing, staining and imaging (Fig. 5A). Quantification of filipin fluorescence showed a decrease in cholesterol with individual treatments, and a synergistic effect when both treatments were administered together (Fig. 5B). We observed that a treatment of 0.05 mM HPβCD and 5 μM DSPE-PEG_2k_ showed a 30% cholesterol clearance, which was further reduced to 50% with 0.05 mM HPβCD and 10 μM–100 μM DSPE-PEG_2k_ treatment. This effect became more pronounced as the concentration of each component was increased. A 75% cholesterol reduction resulted from treatment of 0.25 mM of HPβCD combined with 10 μM DSPE-PEG_2k_, making cholesterol levels comparable to wild-type cells. It has been observed that a dose of 1 mM HPβCD in *Npc1* mutant neuronal cultures or in plasma of human patients elicits a drug response that affects the disease phenotype^8^,^24^. We showed that 1 mM HPβCD reduces cholesterol by ~50%, but a comparable cholesterol reduction can be achieved with 10 μM DSPE and a 4-fold lower concentration of HPβCD (0.05 mM). We observed no toxicity at these concentrations, and only at an exceptionally high treatment combination i.e. 10 mM HPβCD and 500 μM DSPE-PEG_2k_, there was a dramatic reduction in total cell number (Fig. S2).

**Figure 6:**
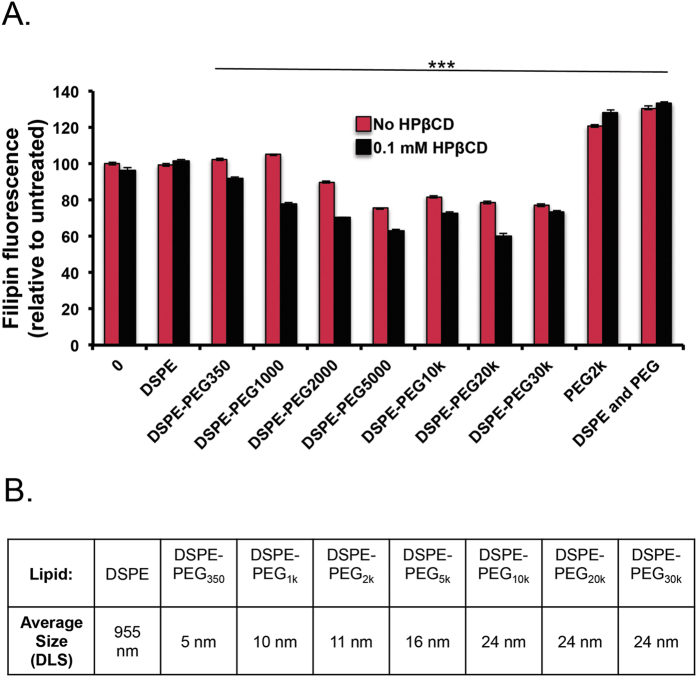
PEG chain length in DSPE-PEG micelles affects cholesterol reduction in *Npc1*^−/−^ cells. (**A**) *Npc1*^−/−^ MEFs were treated with DSPE-PEG micelles (10 μM) with increasing PEG chain length in the presence or absence of low dose HPβCD (0.1 mM). Cells were washed and stained for filipin. (**A**) Longer PEG chain lengths (>2,000 MW) show effective cholesterol reduction when treated with 10 μM. Micelle treatment with 0.1 mM HPBCD additionally reduces cholesterol when administered with DSPE-PEG micelles. (**B**) DLS was used to calculate average diameter of DSPE-PEG micelles, data is reported as distributed by number. Results were tested for statistical significance using one-way ANOVA with Tukey’s multiple comparison test. *P < 0.05, **P < 0.01, ***P < 0.001 and NS = not significant.

PEG-lipids are commercially available with various PEG chain lengths, and we tested a diverse set that included DSPE-PEG_350_, DSPE-PEG_1k_, DSPE-PEG_2k_, DSPE-PEG_5k_, DSPE-PEG_10k_, DSPE-PEG_20k_, and DSPE-PEG_30k_ at 10 μM both with and without 0.1 mM HPβCD (Fig. 6A). DSPE (unconjugated), DSPE-PEG_350_ and DSPE-PEG_1k_ treatments did not decrease cellular cholesterol. On the other hand, DSPE-PEG_2k_, DSPE-PEG_5k_, DSPE-PEG_10k_, DSPE-PEG_20k_, and DSPE-PEG_30k_ showed cholesterol reduction where longer PEG chain lengths performed marginally better relative to shorter chain lengths. The co-treatment of low dose DSPE-PEGs with 0.1 mM HPβCD further reduced cellular cholesterol in most treatments. Interestingly, treatment with PEG_2k_ or combination treatment of PEG_2k_ and DSPE (unconjugated) showed increased cholesterol accumulation in *Npc1*^−/−^ cells (Fig. 6A, S3). We observed that as the PEG chain length increases the size of the micelles also increase from 5 nm (DSPE-PEG_350_) to 24 nm (DSPE-PEG_10–30k_). Micelles with the hydrodynamic size of 16–24 nm, independent of PEG chain length, were capable of equivalent cholesterol efflux suggesting that this size range is important for access to lysosomal pathways (Fig. 6B). We further tested if other polymers with micelle forming capacity can cause cholesterol clearance. Pluronic block copolymers are composed of poly (ethylene oxide) (PEO) and poly(propylene oxide) (PPO) segments, that above CMC self assemble as micelles with hydrophobic PPO cores and hydrophilic PEO shell. These polymers have the ability to solubilize drugs for cellular internalization and have been used extensively to deliver various drugs^25^. Pluronic block copolymers interact with cholesterol subdomains within lipid rafts in cellular membranes and cause membrane fluidization^26^. We tested whether a set of block copolymers including L64, P84, P103 and F127 containing 30, 39.7, 59 and 65.2 number of respective PPO blocks would affect cholesterol efflux. We observed effects on cholesterol clearance with F127 at 1.5 mg/ml, which is an exceptionally high dose. The other polymers had showed toxicity at a high dose in *Npc1*^−/−^ cells (Fig. S4).

**Figure 7:**
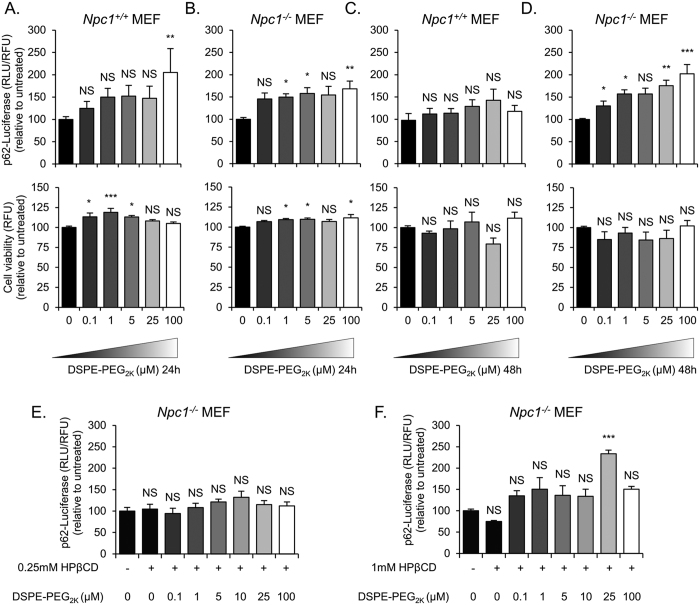
DSPE-PEG treatment slightly reduces autophagic degradation in *Npc1*^+/+^ and *Npc*^−/−^ MEFs (**A,C**) p62-fluc.*Npc1*^+/+^ and, (**B,D**) p62-fluc. *Npc*^−/−^ MEFs were induced to accumulate p62-fluc fusion protein with 1 μg/ml of Doxycycline for 24 hours. Doxycycline was washed from the cells and then they were treated with 0–100 μM DSPE-PEG_2k_ for 24 (**A** and **B** upper panels) or 48 (**C** and **D**, upper panel) hours and autophagic degradation was measured using luciferase intensity. The cells were also analyzed for cell viability (lower panels). (**E,F**) *Npc1*^−/−^ p62-fluc were also treated with a combination of HPβCD (0.25 mM or 1 mM, respectively) and DSPE-PEG (0–100 μM) for 24 hours after being induced for 24 hours with 1 μg/ml of Doxycycline. Results were tested for statistical significance using one-way ANOVA with Tukey’s multiple comparison test. *P < 0.05, **P < 0.01, ***P < 0.001 and NS = not significant.

**Figure 8:**
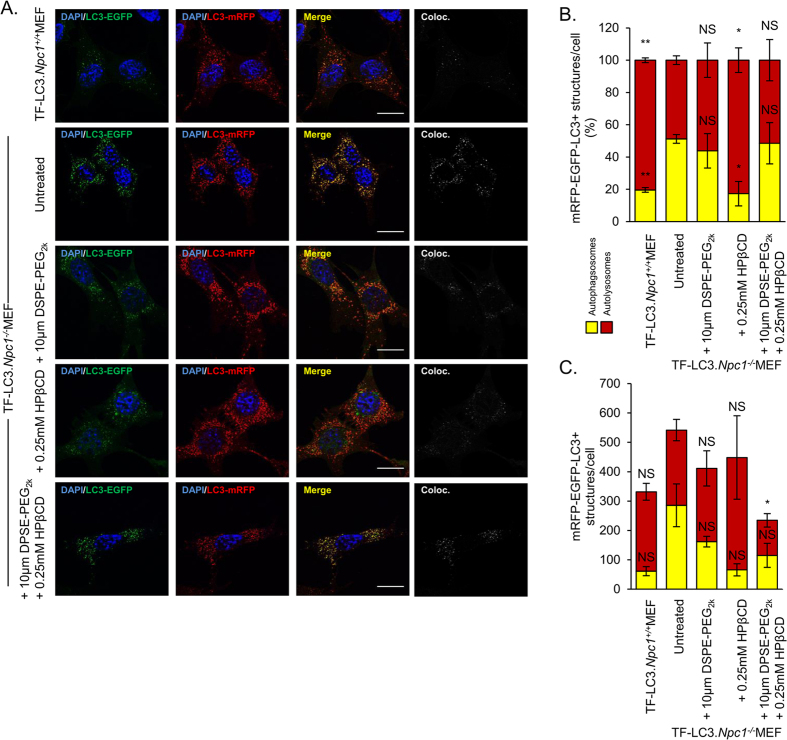
The effect on autophagic flux by DSPE-PEG and HPβCD combination treatment. TF.LC3.*Npc1*^+/+^ and TF.LC3.*Npc1*^−/−^ MEFs were treated for 24 hours with 10 μM DSPE-PEG_2k_, 0.25 mM HPβCD or the combination of the two chemicals. Cells were then fixed, counterstained with DAPI and imaged at 100x magnification. (**A**) Representative images showing EGFP signal (far-left panel), mRFP signal (mid-left panel), merged EGFP and mRFP (mid-right panel) and colocalization of EGFP and mRFP (far-right panel). Scale bar = 20 μm. Images were quantified using ImageJ software. Quantification of microscope images showing the (**B**) number of autophagosomes and autolysosomes as a percentage or as (**C**) total number. Results were tested for statistical significance using two-tailed student’s t-test. *P < 0.05, **P < 0.01, ***P < 0.001 and NS = not significant.

Autophagy, a catabolic process that directs cytosolic components towards the lysosomes for degradation, has been suggested to be dysregulated in lysosomal storage disorders^27^. It has been shown that *Npc1*^−/−^ cells have a block in autophagic flux that leads to accumulation of toxic components within the cells^28^. We evaluated autophagic activity using *Npc1*^−/−^ and *Npc1*^+/+^ MEFs that stably express a doxycycline-inducible firefly luciferase-p62 construct (p62-fluc). p62 acts as a marker for cellular cargo that is specifically degraded by autophagy. Therefore, higher luciferase expression corresponds to lower autophagic degradation. In *Npc1*^+/+^ MEFs there was a little if any retardation in the degradation of p62-fluc with increasing concentrations of DSPE-PEG without any significant effect on cell viability when treated for 24 hours and 48 hours (Fig. 7A,C). We then analyzed the effect on autophagy in *Npc1*^−/−^ MEFs treated with DSPE-PEG. While, we did not observe a change in p62 degradation in 24 hours post treatment (Fig. 7B) a dose-dependent decrease in p62-fluc degradation was seen with DSPE-PEG treatment after 48 hours, without any substantial effect on cell viability (Fig. 7D). We treated *Npc1*^−/−^ MEFs for 24 hours with a combination of 0.25 mM or 1 mM HPβCD and between 0.1 and 100 μM DSPE-PEG. We observed that the combinations at these doses did not substantially exacerbate autophagy (Fig. 7E,F). To further investigate the effect of DSPE-PEG and HPβCD treatment on the autophagic flux we used *Npc1*^−/−^ and *Npc1*^+/+^ MEFs that stably express a tandem fluorescent-tagged LC3 reporter, TF LC3 (mRFP-EGFP-LC3). LC3 is a specific marker of autophagosomes since it is present on autophagosomal membrane from their formation until degradation in autolysosomes^29^. Coupling LC3 with mRFP and EGFP gives us the ability to distinguish between autophagosomes (mRFP^+^/EGFP^+^) and autolysosomes (mRFP^+^/EGFP^−^) as EGFP is acid-labile and therefore its signal is quenched in autolysosomes. We noted an increased number of autophagosomes and decreased number of autolysosomes in the *Npc1*^−/−^ MEFs compared to *Npc1*^+/+^ MEFs (Fig. 8A), suggestive of an impairment in autophagy in NPC1 disorder, as previously described^28^. A combination of 0.25 mM HPβCD and 10 μM DSPE-PEG, compared to 10 μM DSPE-PEG alone had no effect on the percentage of autophagosomes and autolysosomes (Fig. 8B). However, there was a decrease in the overall number of autophagosomes and autolysosomes (Fig. 8C) upon exposure of these admixtures, although this difference did not translate into defects of p62 clearance (Fig. 7), which suggests that this combination may have an effect on the dynamics of autophagic vesicles without exacerbating defects in autophagic cargo clearance. Overall, these studies indicate that low dose combination treatment with these molecules to enable cholesterol efflux, do not worsen the defects in autophagy prevalent in *Npc1*^−/−^ cells.

## Discussion

PEG-conjugated lipids are used extensively in the field of drug delivery as excipients^30^. DSPE-PEGs are biocompatible, biodegradable and have been a component of several nanoparticle platforms for drug or nucleic acid delivery that are undergoing clinical trials^30^,^31^,^32^,^33^. The liposomal formulation Doxil contains DSPE-PEG as one of its constituents and has been clinically approved for use against ovarian cancer and AIDS related Kaposi sarcoma^34^. DSPE anchors PEG to the surface of these liposomes, which forms a protective hydrophilic coating that maintains stability of the nanoparticles. PEGylation of nanoparticles mitigates binding to serum proteins, which provides certain stealth effects, as nanoparticles evade recognition and opsonization by macrophages and prolongs circulation time^17^,^35^,^36^. Tuning PEG density at the surface of nanocarriers allows delivery across biological barriers, e.g. high PEG density assists in traversing mucosal barriers and can also enable widespread distribution in the CNS tissue once injected directly into the brain^37^,^38^. PEG-lipids are amphiphilic by nature and at low concentrations exist as single-coil unimers; above the CMC PEG-lipids form micelles with a core-shell morphology. The hydrophobic core can solubilize hydrophobic drugs, while the shell interacts with the aqueous serum environment^39^. The derivitization of PEG-lipids allows liposomal surface decoration of a repertoire of ligands for the purposes of tissue targeting. These conjugates have been even used to tether ligands on the surface of cells for *ex vivo* therapies^40^. DSPE-PEG micelles have been explored as carriers for diagnostic imaging agents, and have been safely injected into mice with a detected circulation concentration of greater than 1 mM, three orders of magnitude above the CMC^41^. These studies, to the best of our knowledge, for the first time reveal that DSPE-PEG can have the ability to preferentially accumulate inside the late endosomes of *Npc1*^−/−^ cells and remove cholesterol from the endo/lysosomal system.

The NPC1 protein is a cholesterol efflux transporter that shunts cholesterol from the late endosomes to the endoplasmic reticulum^42^. Functional absence of NPC1 results in lipid accumulation in late endosomes/lysosomes. We observed cholesterol aggregates sequestered in the lumen of Rab7 positive late endosomes. Cholesterol aggregation results in cytotoxicity of neurons and hepatocytes due to their high sensitivity towards lipid accumulation^5^. Furthermore, the absence of NPC1 was shown to downregulate caveolae/lipid raft-mediated endocytosis while other entry pathways, such as macropinocytosis and clathrin-mediated endocytosis, remained intact^43^,^44^. Moreover, it was discovered that biodegradable nanoparticles that utilize caveolae/lipid-raft based micro-domains to enter cells fail to internalize and deliver DNA in *Npc1*^−/−^ cells, while LNPs that enter cells using macropinocytosis show a high retention due to defects in their recycling in *Npc1*^−/−^ cells that translate to effective siRNA delivery at very low doses^21^. Interestingly, this study shows similar retention of DSPE-PEG in late endosomes/lysosomes through a striking co-localization with filipin, which suggests that these nanocarriers possibly interact with cholesterol in the closed microenvironment of the late-endosomes and promote cholesterol solubilization. We speculate that within the DSPE-PEG construct, the DSPE serves to integrate into the cell membrane to enter cells and further interacts with the hydrophobic sterol aggregates promoting solubilization while the PEG chains interact with the hydrophilic glycocalyx that surrounds the lumen of the lysosomes^45^, enabling cholesterol efflux from these compartments. DSPE-PEG has been shown to stimulate cholesterol release from synthetic donor liposomes; perhaps such cholesterol release occurs in the multi-vesicular late endosome as well^46^. It is possible that the presence of DSPE-PEG triggers the formation of liposomal structures *in situ* that disassembles cholesterol/lipid aggregates, with the PEG chain lining the surface of the liposome to promote transfer across the lysosomal luminal membrane. PEGs exist in brush or mushroom conformations dependent on chain length, when assembled onto a nanocarrier surface^47^, and whether these conformations form within the confines of the endosomal microenvironment remains to be tested. It is also possible that among the different PEG-lipids, a particular size range may play an important role in targeting nanocarriers precisely to the endosomal compartment that requires cholesterol sequestration.

NPC is a devastating disease for which no treatment is available in the United States. There is a tremendous excitement among scientists for a solution for this disease as preclinical animal models and patients respond positively to HPβCD treatment. Yet, the relatively high dose required to achieve therapeutic response has been associated with hearing loss that occurs due to interaction of HPβCD with prestin, a protein located on outer-hair cells of the cochlea^48^. To overcome these issues of efficacy, new polymers such as Pluronic/β-cyclodextrin polyrotaxanes have been synthesized to release multiple cyclodextrin molecules in the reducing endosomal environment^49^,^50^,^51^. Unfortunately these new compounds will require extensive preclinical and clinical testing prior to translational development. HPβCD, at high doses, exacerbates autophagy defects; on the other hand, repeated administration of PEG has been associated with complement activation^28^,^52^ and perhaps by combining DSPE-PEG and HPβCD a synergistic response at low doses can promote cholesterol egress without causing toxicity. It has been observed in *Npc1*^−/−^ cats that a 120 mg of intra-cisternal dose of HPβCD increased survival from 22 weeks to 76 weeks to 4 years^12^, and it is therefore, tempting to speculate that injecting admixtures of cyclodextrin with DSPE-PEG, directly into the brain will lower the dose necessary for survival and decrease the substantial side effects of HPβCD, otherwise observed in clinical and preclinical models of NPC disorder.

Liposomes with increasing amounts of PEG-lipids were developed for encapsulation and co-delivery of HPβCD, and we observed that the liposomal size decreased from roughly 100 nm to 10–20 nm as the PEG-lipid molar ratio increased. Nanoparticles transition from an aqueous core encapsulated in a lipid bilayer to a micelle with a hydrophobic core with increasing molar ratios of DSPE-PEG, thus decreasing the encapsulation efficiency of the hydrophilic drug. We observed that when treatments were normalized for HPβCD concentrations and constant lipid concentrations, the nanocarriers with higher DSPE-PEG content resulted in substantial cholesterol clearance, and DSPE-PEG micelles were the most potent. PEG-lipid micelles or liposomes with high amounts of DSPE-PEG might serve as bioactive drug delivery agents for treatment of NPC. We were further able to test whether other polymers have the ability to egress cholesterol. We tested a set of Pluronic block copolymers that have the capability to self-assemble as micelles. Pluronic micelles utilize clathrin-mediated endocytosis to access endo/lysosomes and modulate cholesterol fluidity to inhibit drug efflux transport, activate gene expression, and induce dose-dependent hyperlipidemia^25^,^53^. Among the materials tested, Pluronic F127 showed an effect at very high concentrations, suggesting that some of these surfactants might cause non-specific cholesterol clearance.

There are several new strategies for potential therapeutics against NPC disease that may require nanocarrier based drug delivery systems. Vironosotat, a histone deacetylase inhibitor (HDACi) has been shown to clear cholesterol in human patient fibroblasts carrying the NPC1^I1061T^ mutation^54^,^55^. HDACi inhibitors enhance transcriptional activity; overexpression partially compensated for the NPC1^I1061T^ mutation and acts as a chaperone to untangle mis-folded protein. HPβCD has recently been used to solubilize the hydrophobic vironosat, and when administered subcutaneously to NPC1^I1061T^ mutant mice, substantially improved life span^56^. However, this formulation consists of high amounts of cyclodextrin and 5% DMSO, a toxic organic solvent that could limit clinical applicability^56^. Carbamazepine an autophagy inducer used to overcome the autophagy blockade^57^ and remove toxic cytosolic debris, will be an important ingredient in the formulation for treatment of NPC^28^. PEG-lipid micelles can entrap hydrophobic drugs, such as vironostat or carbamazepine in the core, and when formulated with cyclodextrin could result in a potent drug combination against NPC. As new variants of cyclodextrin that have different degree of substitutions are identified for improved efficacy and reduced ototoxicity in NPC disease, these agents can be replaced in the aforementioned drug cocktail^58^. Additionally genetic editing and gene delivery approaches have been used to correct mutated genes, including *Npc1*, which offers a precise treatment and potential cure for rare genetic disorders and can benefit from non-viral delivery technologies^19^,^59^. Finally, traversing the blood brain barrier is required for successful translation of this technology, and we anticipate modification of these nanocarriers with multivalent targeting ligands for brain delivery^60^. Potentially attaching new brain shuttle technologies that have shown to enhance brain penetration in different animal models, including non-human primates, or temporary opening of the blood- brain barrier will enable brain targeting^61^,^62^. This information serves as a blueprint to further develop PEG-lipid based bioactive nanocarriers with brain targeting capability that can exploit trafficking defects to site-specifically deliver drugs or their combinations for effective treatment of NPC. Taken together, nanocarriers can serve as a platform that can effectively deliver small molecules, genes and perhaps imaging agents for treatment and diagnosis of a wide variety of other rare lysosomal storage disorders.

## Materials and Methods

### Assembly of Liposomes or Micelles

Liposomes were formed using the thin film rehydration method. Briefly HSPC (Avanti Polar Lipids), DSPE-PEG_2k_ (NOF America) or a combination, were dissolved in ethanol at 20 mM. To make thin films, 2 μMol of lipid was dried in 20 mL glass scintillation vial (approximately 1 in^2^) under vacuum and rehydrated by sonication for 20 mins at 40 °C in a bath sonicator. Liposomes were formed in 200 μL of 400 mM HPβCD (ACROS Organics) in PBS. Clean DSPE-PEG_2k_ micelles were assembled in 200 μL Opti-MEM and cooled on ice until further use. Liposomes were washed twice in PBS to remove unencapsulated HPβCD using a centrifugal filter concentrator (Amicon Ultra−15 10 k MWCO) for 10 minutes at 17 k × G. Assembled micelles (without HPβCD) were diluted as necessary to be used in cell treatment studies. Additional lipid micelles, including DSPE, DSPE-PEG_350_, DSPE-PEG_1k_, DSPE-PEG_5k_ or DSPE-PEG_2k_-CF (Avanti-Polar lipids) and DSPE-PEG_10k_, DSPE-PEG_20k_, DSPE-PEG_30k_, (Creative PEGWorks) were formed from lipid stocks stored in chloroform. Thin films (100 nmoles lipid) were formed as described above and hydrated in 100 μL Opti MEM to make 1 mM stock solutions. For PEG_2k_ a 10 μM solution was made from powder rehydrated in Opti MEM, and diluted to desired concentrations. Pluronics P84 (Sigma Aldrich), L64, F127 and P103 (BASF SE) were diluted from stock solutions (150 mg/mL) in Opti MEM for cell treatments as described above.

### Materials Analysis

Liposomes or micelles were confirmed using dynamic light scattering (Malvern Zetasizer) in PBS and average size is reported based on a number distribution. Transmission Electron Microscopy (TEM) was performed on an FEI Tecnai F-20 TEM operating at 200 kV. Samples of liposomes or micelles were prepared by drop casting onto a carbon coated copper grid (Ted Pella), and allowed to air dry. HPβCD was measured using an adapted LC-ESI-MS/MS method^63^. Briefly, washed liposomes were digested in methanol and subjected to high-performance liquid chromatography (HPLC)-tandem mass spectrometry using a 75 × 2 mm C18 3 μm particle LC column (Phenomenex Torrance, CA) coupled to a SCIEX 4000 QTRAP LC/MS/MS equipped with a electrospray ionization (ESI) source operating in the positive mode. The HPLC column temperature was kept at 40 °C. Analyte and internal standard (methyl-β-cyclodextrin) were resolved using a gradient method with solvents A (methanol at 0.1% formic acid) and B (100 mM ammonium formate) at a flow rate of 0.5 ml/min changed as follows: 5% to 95% B over 1 minute, held at 95% B for 1.5 minutes and equilibrated at 5% B for 2.5 minutes. The ESI ion source parameters were as follows: 48 V collision energy, 30 units curtain gas, 5000 V ion spray, 450 C temperature, 40 units gas 1 and 50.00 gas 2. Multiple reaction monitoring mass transitions scanned for 100 msec were m/z 1307 → 367.4 for methyl-β-cyclodextrin, and m/z 1385 → 383.4 and 1443 → 383.4 for HPβCD. HPβCD and internal standard had a retention time of approximately 1.7 minutes. Calibration curves for quantification were created using HPβCD standard across the concentration range 0.05–500 μg/ml with methyl-β-cyclodextrin at 25 μg/mL. The area ratio of analyte to internal standard was plotted against the nominal concentration for calibrators and a quadratic regression analysis was used to calculate the concentration in unknown samples.

### Imaging

*Npc1*^−/−^ and *Npc1*^+/+^ immortalized mouse embryonic fibroblasts (MEFs) (kind gifts from the Langer Lab at MIT) were cultured in DMEM/10% FBS at 37 °C. Cells were plated into a 96 well plate (4 k cell/well with 100 μL media) and allowed to settle for 24 hours. Intracellular staining of cholesterol in these cells was performed using filipin staining of paraformaldehyde fixed cells. Cells were washed in PBS and 2 mM Glycine in PBS for 10 minutes, prior to staining with 0.05 mg/mL, filipin complex (Sigma Aldrich) in 10% FBS for 2 hours. Cells were then washed in PBS three times and stored in 100 μL PBS with 1x HCS NuclearMask Deep Red Stain (Life Technologies) at 4 °C for further analysis. *Npc1*^−/−^ cells were treated with drug (HPβCD), PEG-lipid or a combination of drug with PEG-lipid, dissolved in Opti-MEM (Thermo Fisher). After a 24-hour incubation, media was removed, cells were washed in PBS and fixed in 4% paraformaldehyde at room temperature for 20 minutes, followed by filipin staining. Reduction of filipin staining from the lysosomal stores of *Npc1*^−/−^ cells is referred as cholesterol efflux. For Rab7-GFP (marker of late endosomes) experiments, *Npc1*^+/+^ or *Npc1*^−/−^ cells were each transfected with Rab7-GFP using a baculovirus (Bacmam 2.0) for 18 hours, fixed and filipin stained as described, and imaged. For uptake and localizationstudies,1,2-distearoyl-*sn*-glycero-3-phosphoethanolamine-N-[poly(ethylene glycol)-2000-N′-carboxyfluorescein] (Avanti Polar Lipids) (DSPE-PEG-CF) were assembled into micelles (described below) and incubated with cells for specified time points prior to fixing and filipin staining. High throughput imaging was performed using either an automated high-throughput fluorescence microscope (Olympus scanR) at 20X with at least 16 images/well were analyzed using the Olympus scanR analysis software. In select cases, EVOS FL Auto Cell imaging system (Invitrogen) 20X or 40X was utilized. All experiments were performed in triplicates and errors are reported as S.E.M.

### Total cellular cholesterol from cell lysates

Amplex Red cholesterol assay (Amplex Red Cholesterol Assay Kit, ThermoFisher) was performed according to manufacturers instructions and used to quantify total cellular cholesterol from cell lysates, which was normalized to total protein content using the Bradford Assay. Briefly, after drug treatments, cells were washed with PBS and lysed with Cholesterol Mammalian Protein Extraction Reagent (ThermoFisher Cat # 78501). The cells were frozen for 10 mins at −80 °C and plates were thawed for 30 mins at 37 °C. The cell lysates were treated with Amplex red reagent (prepared using Manufacturers protocol) in presence of Halt Protease inhibitor (Halt™ Protease and Phosphatase Inhibitor Cocktail, EDTA-free (100X), ThermoFisher Cat # 78445). Cell lysates were incubates at room temperature for 1 hr and fluorescence (Ex/Em = 540/590) was measured using Tecan Microplate reader (Tecan). The cell lysates were treated with Bradford reagent (Pierce™ Coomassie (Bradford) Protein Assay Kit, (ThermoFisher) and their absorbance (595 nm) was measured. Standard curves were plotted using known concentrations of Cholesterol (Amplex Red assay) and Bovine Serum Albumin (Bradford) to interpolate cell lysates based data. All experiments were performed in triplicates and errors are reported as S.E.M.

### Cloning and generation of stable cell lines

#### p62-Lucifearse expressing Npc1 stable cell lines

*Npc1*^+/+^ and *Npc1*^−/−^ MEFs were transduced with a doxycycline inducible p62-firefly luciferase (p62-fluc) construct that also had a puromycin resistance gene were maintained in 1 μg/ml puromycin (Sigma-Aldrich P8833). Briefly, the doxycycline inducible pCW57.1 backbone was purchased from Addgene (41393). Gibson assembly was used to insert firefly luciferase (Fluc) and p62 into the pCW57.1 backbone. Fluc, p62 and pCW57.1 were PCR amplified using pfu polymerase (Life technologies) and Gibson assembly primers with 20 bp overhangs. PCR products were separated by agarose gel electrophoreses, and fragments excised and purified before use in DNA assembly reaction with the NEBuilder HiFi DNA Assembly kit (New England Biolabs) according to the manufacturer’s instructions. After the assembly, the reaction mix was transformed into NEB3040 NEB Stable Competent *E. coli* (New England Biolabs). After 24 h growth at 31 °C the plasmid was extracted using QIAprep Spin Miniprep Kit (Qiagen). The plasmid was sequenced to confirm correct insertion of Fluc-p62. To generate *Npc1*^+/+^ and *Npc1*^−/−^ MEFs stably expressing doxycline inducible Fluc-p62, HEK293FT cells were transfected with Fluc-p62-pCW57.1 and the ViraPower Packaging Mix (Invitrogen) to produce lentivirus. *Npc1*^+/+^ and *Npc1*^−/−^ MEFs were transduced with the obtained lentiviral stock followed by selection with 1 μg/ml puromycin.

Gibson primers: pCW57.1FW-AGCATCCCCCGCCGTTGTGATTCTTGTACAAAGTGGTTTAGTAATGAACCGGTCCA pCW57.1 REV-ATGTTTTTGGCGTCTTCCATTGATGCTAGCCAATTCTCCAGGCGATC firefly luciferaseFW-TGGAGAATTGGCTAGCATCAATGGAAGACGCCAAAAACATAAAGAAAGG firefly luciferase REV-TTCACGGTGAGCGACGCCATCACGGCGATCTTTCCGCCCT p62 FWD-GAAAGATCGCCGTGATGGCGTCGCTCACCGTGAAGGCCTACCTTC p62 REV-TAAACCACTTTGTACAAGAATCACAACGGCGGGGGATGCTTTGAATACTGGA.

#### Tandem Fluorescent LC3 expressing Npc1 stable cell lines

*Npc1*^+/+^ and *Npc*^−/−^ MEFs were transduced with a tandem-fluorescent LC3 reporter (TF-LC3 (mRFP-EGFP-LC3)) which also contained a puromycin resistance gene. MEFs were selected with 1 μg/ml of puromycin. After 1 week of selection the cells were plated as single cells in a 96 well plate to generate monoclonal TF-LC3 lines. pLenti-Synapsin-hChR2(H134R)-EYFP-WPRE (purchased from Addgene (20945)) was digested with PacI and EcoRI enzymes. TF-LC3 and IRES-PURO were amplified using PCR with Q5 HighFidelity DNA Polymerase (NEB) using Gibson Assembly primers, which contained 15bp overhangs. Purified amplicons were mixed and assembled using Gibson Assembly Master Mix (NEB). The assembled DNA was transformed into NEB® 5-alpha Competent *E. coli* (NEB). DNA was amplified in *E. coli* and sequenced to check for correct insertion of the TF-LC3 and the puromycin resistance gene. Then plasmid DNA was transfected into 293T cells with lentiviral packaging plasmids (tat, rev, gag/pol and vsv-g). Lentiviral particles were collected after 24 hours and used to transduce *Npc1*^+/+^ and *Npc*^−/−^ MEFs.

Gibson primers:TF-LC3-F – AGATCCAGTTTGGTTAATACCATGGCCTTCTCCGAG TF-LC3-R – CCATCTTCCTGTCACAAGCATGGCTC IRES-PURO-F – TGTGACAGGAAGATGGCGATTAGAGATCC IRES-PURO-R – CGATAAGCTTGATATCGGATTTAGGCACCGGGCTTGC.

### Luciferase Assay

First, the p62-fluc.*Npc1*^+/+^ and p62-fluc.*Npc1*^−/−^ MEFs were plated into white 96 well plates (2 k cell/well with 100 μL media) and allowed to settle for 24 hours. Next, the MEFs were treated with 1 μg/ml doxycycline (Sigma-Aldrich 33429) for 24 hours before being washed off 3 times with PBS. Subsequently, the specified drug or a combination of drugs was dissolved in Opti-MEM (Thermo Fisher) and all of media was removed from cells and replaced with DSPE-PEG_2k_ containing media and incubated for 24 hours. MEFs to be treated for 48 hours with the micelles were aspirated after 24 hours and fresh DSPE-PEG_2k_ media was added for the final 24 hours. After 24 hours or 48 hours of drug treatment, MEFs were analyzed using ONE-Glo™ + Tox Luciferase Reporter and Cell Viability Assay (Promega) by following the manufacturer’s protocol.

### TF.LC3 analysis

Analysis of autophagosome maturation using mRFP-EGFP-LC3 (TF-LC3) reporter was performed in the following way. TF-LC3.*Npc1*^+/+^ and TF-LC3.*Npc*^−/−^ MEFs were plated onto 9 mm glass coverslips coated with 0.2% gelatin (Sigma) at a density of 25000 per coverslip. The following day the cells were treated with for 24 hours with 10 μM DSPE-PEG2k, 0.25 mM HPβCD or the combination of the two chemicals. After 24 hours the cells were washed with PBS and then fixed with 4% methanol free-paraformaldehyde (Thermo Fisher) for 15 minutes at room temperature. The formaldehyde was washed off twice with PBS and then the cells were quenched with 100 mM glycine (Sigma) for 10 minutes. Next, the cells were permeabilized with 0.5% Triton X-100 (Sigma) for 10 minutes. Subsequently, cells were washed twice with PBS and then counter stained with 1x DAPI for 5 minutes at room temperature. The DAPI was washed off twice with PBS and then the coverslips were mounted onto Prolong Diamond Anti-fade reagent (Life Technologies). Mounted slides were cured overnight and then imaged on a Carl Zeiss LSM 780 confocal microscope at 100x magnification. Images were processed using ImageJ software.

## Additional Information

**How to cite this article**: Brown, A. *et al.* PEG-lipid micelles enable cholesterol efflux in Niemann-Pick Type C1 disease-based lysosomal storage disorder. *Sci. Rep.* **6**, 31750; doi: 10.1038/srep31750 (2016).

## Supplementary Material

Supplementary Information

## References

[b1] Carstea E. D. *et al.* Niemann-Pick C1 Disease Gene: Homology to Mediators of Cholesterol Homeostasis. Science 277, 228–231 (1997). 9211849 10.1126/science.277.5323.228

[b2] Vanier M. T. Niemann-Pick disease type C. Orphanet J. Rare Dis. 5, 16 (2010). 20525256 10.1186/1750-1172-5-16 PMC2902432

[b3] Ikonen E. Cellular cholesterol trafficking and compartmentalization. Nat. Rev. Mol. Cell Biol. 9, 125–138 (2008). 18216769 10.1038/nrm2336

[b4] Ottinger E. A. *et al.* Collaborative development of 2-hydroxypropyl-β-cyclodextrin for the treatment of Niemann-Pick type C1 disease. Curr. Top. Med. Chem. 14, 330–339 (2014). 24283970 10.2174/1568026613666131127160118 PMC4048128

[b5] Walkley S. U. & Suzuki K. Consequences of NPC1 and NPC2 loss of function in mammalian neurons. Biochim. Biophys. Acta 1685, 48–62 (2004). 15465426 10.1016/j.bbalip.2004.08.011

[b6] Szente L. & Szejtli J. Highly soluble cyclodextrin derivatives: chemistry, properties, and trends in development. Adv. Drug Deliv. Rev. 36, 17–28 (1999). 10837706 10.1016/s0169-409x(98)00092-1

[b7] Kilsdonk E. P. C. *et al.* Cellular Cholesterol Efflux Mediated by Cyclodextrins. J. Biol. Chem. 270, 17250–17256 (1995). 7615524 10.1074/jbc.270.29.17250

[b8] Peake K. B. & Vance J. E. Normalization of Cholesterol Homeostasis by 2-Hydroxypropyl-β-cyclodextrin in Neurons and Glia from Niemann-Pick C1 (NPC1)-deficient Mice. J. Biol. Chem. 287, 9290–9298 (2012). 22277650 10.1074/jbc.M111.326405 PMC3308731

[b9] Davidson C. D. *et al.* Chronic Cyclodextrin Treatment of Murine Niemann-Pick C Disease Ameliorates Neuronal Cholesterol and Glycosphingolipid Storage and Disease Progression. PLoS One 4 (2009). 10.1371/journal.pone.0006951 PMC2736622 19750228

[b10] Camargo F. *et al.* Cyclodextrins in the treatment of a mouse model of Niemann-Pick C disease. Life Sci. 70, 131–142 (2001). 11787939 10.1016/s0024-3205(01)01384-4

[b11] Ramirez C. M. *et al.* Weekly Cyclodextrin Administration Normalizes Cholesterol Metabolism in Nearly Every Organ of the Niemann-Pick Type C1 Mouse and Markedly Prolongs Life. Pediatr. Res. 68, 309–315 (2010). 20581737 10.1203/PDR.0b013e3181ee4dd2 PMC3065173

[b12] Vite C. H. *et al.* Intracisternal cyclodextrin prevents cerebellar dysfunction and Purkinje cell death in feline Niemann-Pick type C1 disease. Sci. Transl. Med. 7, 276ra26 (2015). 10.1126/scitranslmed.3010101 PMC4415615 25717099

[b13] Maarup T. J. *et al.* Intrathecal 2-hydroxypropyl-beta-cyclodextrin in a single patient with Niemann-Pick C1. Mol. Genet. Metab. 116, 75–79 (2015). 26189084 10.1016/j.ymgme.2015.07.001 PMC4633280

[b14] Torchilin V. P. Recent advances with liposomes as pharmaceutical carriers. Nat. Rev. Drug Discov. 4, 145–160 (2005). 15688077 10.1038/nrd1632

[b15] Szoka F. & Papahadjopoulos D. Procedure for preparation of liposomes with large internal aqueous space and high capture by reverse-phase evaporation. Proc. Natl. Acad. Sci. USA. 75, 4194–4198 (1978). 279908 10.1073/pnas.75.9.4194 PMC336078

[b16] Allen T. M. & Cullis P. R. Liposomal drug delivery systems: from concept to clinical applications. Adv. Drug Deliv. Rev. 65, 36–48 (2013). 23036225 10.1016/j.addr.2012.09.037

[b17] Klibanov A. L., Maruyama K., Torchilin V. P. & Huang L. Amphipathic polyethyleneglycols effectively prolong the circulation time of liposomes. FEBS Lett. 268, 235–237 (1990). 2384160 10.1016/0014-5793(90)81016-h

[b18] Pardridge W. M. Blood-brain barrier drug targeting: the future of brain drug development. Mol. Interv. 3, 90–105 (2003). 14993430 10.1124/mi.3.2.90

[b19] Yin H. *et al.* Non-viral vectors for gene-based therapy. Nat. Rev. Genet. 15, 541–555 (2014). 25022906 10.1038/nrg3763

[b20] Guo X. & Huang L. Recent advances in nonviral vectors for gene delivery. Acc. Chem. Res. 45, 971–979 (2012). 21870813 10.1021/ar200151m PMC3240701

[b21] Sahay G. *et al.* Efficiency of siRNA delivery by lipid nanoparticles is limited by endocytic recycling. Nat. Biotechnol. 31, 653–658 (2013). 23792629 10.1038/nbt.2614 PMC3814166

[b22] Vanier M. T. & Latour P. Laboratory diagnosis of Niemann-Pick disease type C: the filipin staining test. Methods Cell Biol. 126, 357–375 (2015). 25665455 10.1016/bs.mcb.2014.10.028

[b23] Rujoi M., Pipalia N. H. & Maxfield F. R. Cholesterol Pathways Affected by Small Molecules That Decrease Sterol Levels in Niemann-Pick Type C Mutant Cells. PLOS ONE 5, e12788 (2010). 20877719 10.1371/journal.pone.0012788 PMC2943465

[b24] Tanaka Y. *et al.* Influence of Npc1 genotype on the toxicity of hydroxypropyl-β-cyclodextrin, a potentially therapeutic agent, in Niemann–Pick Type C disease models. Mol. Genet. Metab. Rep. 1, 19–30 (2014). 10.1016/j.ymgmr.2013.12.003 PMC5121301 27896072

[b25] Kabanov A. V., Batrakova E. V. & Alakhov V. Y. Pluronic® block copolymers as novel polymer therapeutics for drug and gene delivery. J. Controlled Release 82, 189–212 (2002). 10.1016/s0168-3659(02)00009-3 12175737

[b26] Batrakova E. V. & Kabanov A. V. Pluronic block copolymers: evolution of drug delivery concept from inert nanocarriers to biological response modifiers. J. Control. Release Off. J. Control. Release Soc. 130, 98–106 (2008). 10.1016/j.jconrel.2008.04.013 PMC2678942 18534704

[b27] Lieberman A. P. *et al.* Autophagy in lysosomal storage disorders. Autophagy 8, 719–730 (2012). 22647656 10.4161/auto.19469 PMC3378416

[b28] Sarkar S. *et al.* Impaired autophagy in the lipid-storage disorder Niemann-Pick type C1 disease. Cell Rep. 5, 1302–1315 (2013). 24290752 10.1016/j.celrep.2013.10.042 PMC3957429

[b29] Kimura S., Noda T. & Yoshimori T. Dissection of the autophagosome maturation process by a novel reporter protein, tandem fluorescent-tagged LC3. Autophagy 3, 452–460 (2007). 17534139 10.4161/auto.4451

[b30] Che J., Okeke C. I., Hu Z.-B. & Xu J. DSPE-PEG: a distinctive component in drug delivery system. Curr. Pharm. Des. 21, 1598–1605 (2015). 25594410 10.2174/1381612821666150115144003

[b31] MacLachlan I. & Cullis P. Diffusible-PEG-lipid stabilized plasmid lipid particles. Adv. Genet. 53, 157–188 (2005). 16240993

[b32] Dong Y. *et al.* Lipopeptide nanoparticles for potent and selective siRNA delivery in rodents and nonhuman primates. Proc. Natl. Acad. Sci. USA. 111, 3955–3960 (2014). 24516150 10.1073/pnas.1322937111 PMC3964096

[b33] Kauffman K. J., Webber M. J. & Anderson D. G. Materials for non-viral intracellular delivery of messenger RNA therapeutics. J. Control. Release Off. J. Control. Release Soc. doi: 10.1016/j.jconrel.2015.12.032 (2015). 26718856

[b34] Gabizon A. *et al.* Prolonged circulation time and enhanced accumulation in malignant exudates of doxorubicin encapsulated in polyethylene-glycol coated liposomes. Cancer Res. 54, 987–992 (1994). 8313389

[b35] Immordino M. L., Dosio F. & Cattel L. Stealth liposomes: review of the basic science, rationale, and clinical applications, existing and potential. Int. J. Nanomedicine 1, 297–315 (2006). 17717971 PMC2426795

[b36] Gref R. *et al.* Biodegradable long-circulating polymeric nanospheres. Science 263, 1600–1603 (1994). 8128245 10.1126/science.8128245

[b37] Suk J. S., Xu Q., Kim N., Hanes J. & Ensign L. M. PEGylation as a strategy for improving nanoparticle-based drug and gene delivery. Adv. Drug Deliv. Rev. 99, 28–51 (2016). 26456916 10.1016/j.addr.2015.09.012 PMC4798869

[b38] Nance E. A. *et al.* A dense poly(ethylene glycol) coating improves penetration of large polymeric nanoparticles within brain tissue. Sci. Transl. Med. 4, 149ra119 (2012). 10.1126/scitranslmed.3003594 PMC3718558 22932224

[b39] Gill K. K., Kaddoumi A. & Nazzal S. PEG-lipid micelles as drug carriers: physiochemical attributes, formulation principles and biological implication. J. Drug Target. 23, 222–231 (2015). 25547369 10.3109/1061186X.2014.997735

[b40] Takafuji Y. *et al.* Factors influencing the surface modification of mesenchymal stem cells with fluorescein-pegylated lipids. Biol. Pharm. Bull. 36, 1731–1738 (2013). 24189418 10.1248/bpb.b13-00291

[b41] Beilvert A. *et al.* A tyrosine PEG-micelle magnetic resonance contrast agent for the detection of lipid rich areas in atherosclerotic plaque. Magn. Reson. Med. Off. J. Soc. Magn. Reson. Med. Soc. Magn. Reson. Med. 62, 1195–1201 (2009). 10.1002/mrm.22103 PMC2829093 19780153

[b42] Pfisterer S. G., Peränen J. & Ikonen E. LDL-cholesterol transport to the endoplasmic reticulum: current concepts. Curr. Opin. Lipidol. 27, 282–287 (2016). 27054443 10.1097/MOL.0000000000000292 PMC4888931

[b43] Rappaport J., Manthe R. L., Solomon M., Garnacho C. & Muro S. A Comparative Study on the Alterations of Endocytic Pathways in Multiple Lysosomal Storage Disorders. Mol. Pharm. doi: 10.1021/acs.molpharmaceut.5b00542 (2016). PMC4936955 26702793

[b44] Eltoukhy A. A., Sahay G., Cunningham J. M. & Anderson D. G. Niemann-Pick C1 affects the gene delivery efficacy of degradable polymeric nanoparticles. ACS Nano 8, 7905–7913 (2014). 25010491 10.1021/nn501630h PMC4148171

[b45] Li J., Deffieu M. S., Lee P. L., Saha P. & Pfeffer S. R. Glycosylation inhibition reduces cholesterol accumulation in NPC1 protein-deficient cells. Proc. Natl. Acad. Sci. USA. 112, 14876–14881 (2015). 26578804 10.1073/pnas.1520490112 PMC4672801

[b46] Janout V., Daly T. A., Cline L. L., Kulp L. J. & Regen S. L. Stimulated release of cholesterol from liposomal membranes by a PEGylated phospholipid. Bioconjug. Chem. 23, 336–339 (2012). 22372891 10.1021/bc200669e

[b47] Perry J. L. *et al.* PEGylated PRINT Nanoparticles: The Impact of PEG Density on Protein Binding, Macrophage Association, Biodistribution, and Pharmacokinetics. Nano Lett. 12, 5304–5310 (2012). 22920324 10.1021/nl302638g PMC4157665

[b48] Takahashi S. *et al.* Susceptibility of outer hair cells to cholesterol chelator 2-hydroxypropyl-β-cyclodextrine is prestin-dependent. Sci. Rep. 6, 21973 (2016). 26903308 10.1038/srep21973 PMC4763217

[b49] Mondjinou Y. A. *et al.* Synthesis of 2-hydroxypropyl-β-cyclodextrin/pluronic-based polyrotaxanes via heterogeneous reaction as potential Niemann-Pick type C therapeutics. Biomacromolecules 14, 4189–4197 (2013). 24180231 10.1021/bm400922a PMC4314287

[b50] Tamura A. & Yui N. Lysosomal-specific cholesterol reduction by biocleavable polyrotaxanes for ameliorating Niemann-Pick type C disease. Sci. Rep. 4, 4356 (2014). 24619155 10.1038/srep04356 PMC3950578

[b51] Tamura A. & Yui N. β-Cyclodextrin-threaded biocleavable polyrotaxanes ameliorate impaired autophagic flux in Niemann-Pick type C disease. J. Biol. Chem. 290, 9442–9454 (2015). 25713067 10.1074/jbc.M115.636803 PMC4392250

[b52] Chanan-Khan A. *et al.* Complement activation following first exposure to pegylated liposomal doxorubicin (Doxil®): possible role in hypersensitivity reactions. Ann. Oncol. 14, 1430–1437 (2003). 12954584 10.1093/annonc/mdg374

[b53] Batrakova E. V. & Kabanov A. V. Pluronic block copolymers: evolution of drug delivery concept from inert nanocarriers to biological response modifiers. J. Control. Release Off. J. Control. Release Soc. 130, 98–106 (2008). 10.1016/j.jconrel.2008.04.013 PMC2678942 18534704

[b54] Pipalia N. H. *et al.* Histone deacetylase inhibitor treatment dramatically reduces cholesterol accumulation in Niemann-Pick type C1 mutant human fibroblasts. Proc. Natl. Acad. Sci. USA. 108, 5620–5625 (2011). 21436030 10.1073/pnas.1014890108 PMC3078401

[b55] Helquist P., Maxfield F. R., Wiech N. L. & Wiest O. Treatment of Niemann–Pick Type C Disease by Histone Deacetylase Inhibitors. Neurotherapeutics 10, 688–697 (2013). 24048860 10.1007/s13311-013-0217-2 PMC3805865

[b56] Alam M. S., Getz M. & Haldar K. Chronic administration of an HDAC inhibitor treats both neurological and systemic Niemann-Pick type C disease in a mouse model. Sci. Transl. Med. 8, 326ra23 (2016). 10.1126/scitranslmed.aad9407 26888431

[b57] Sarkar S., Maetzel D., Korolchuk V. I. & Jaenisch R. Restarting stalled autophagy a potential therapeutic approach for the lipid storage disorder, Niemann-Pick type C1 disease. Autophagy 10, 1137–1140 (2014). 24879158 10.4161/auto.28623 PMC4091173

[b58] Davidson C. D. *et al.* Efficacy and ototoxicity of different cyclodextrins in Niemann-Pick C disease. Ann. Clin. Transl. Neurol. 3, 366–380 (2016). 27231706 10.1002/acn3.306 PMC4863749

[b59] Maetzel D. *et al.* Genetic and Chemical Correction of Cholesterol Accumulation and Impaired Autophagy in Hepatic and Neural Cells Derived from Niemann-Pick Type C Patient-Specific iPS Cells. Stem Cell Rep. 2, 866–880 (2014). 10.1016/j.stemcr.2014.03.014 PMC4050353 24936472

[b60] Wiley D. T., Webster P., Gale A. & Davis M. E. Transcytosis and brain uptake of transferrin-containing nanoparticles by tuning avidity to transferrin receptor. Proc. Natl. Acad. Sci. USA. 110, 8662–8667 (2013). 23650374 10.1073/pnas.1307152110 PMC3666717

[b61] Yu Y. J. *et al.* Therapeutic bispecific antibodies cross the blood-brain barrier in nonhuman primates. Sci. Transl. Med. 6, 261ra154–261ra154 (2014). 10.1126/scitranslmed.3009835 25378646

[b62] Kroll R. A. & Neuwelt E. A. Outwitting the Blood-Brain Barrier for Therapeutic Purposes: Osmotic Opening and Other Means: *Neurosurgery* 42, 1083–1099 (1998). 10.1097/00006123-199805000-00082 9588554

[b63] Jiang H. *et al.* Development and validation of sensitive LC-MS/MS assays for quantification of HP-β-CD in human plasma and CSF. J. Lipid Res. 55, 1537–1548 (2014). 24868096 10.1194/jlr.D050278 PMC4076072

