# Supplementary material for: PEG-lipid micelles enable cholesterol efflux in Niemann-Pick Type C1 disease-based lysosomal storage disorder
Source: Sci Rep. 2016 Aug 30;6:31750. doi: 10.1038/srep31750 (PMC5004151; doi:10.1038/srep31750)

Supplementary information:

PEG-lipid based micelles cause cholesterol efflux in Niemann Pick Type C-1 disease based lysosomal storage disorder

Anna Brown, Siddharth Patel, Carl Ward, Anna Lorenz, Mauren Ortiz, Allison DuRoss, Fabian Wieghardt, Amanda Esch, Elsje. G. Otten, Laura M. Heiser, Viktor .I. Korolchuk, Conroy Sun, Sovan Sarkar, Gaurav Sahay

Fig. S1 Characterization and efficacy of different liposomal nanocarriers with increasing PEG-lipid content.

Nanocarriers composed of differing ratios of HSPC and DSPE-PEG_{2k} were assembled in the presence of HPβCD A. TEM images of 95 %, 50 % and 25 % HSPC (Scale bar represents 200 nm) of these materials were measured for size and morphology characterization B. DLS of these set of liposomes was measured to characterize size and high-performance liquid chromatography (HPLC)-tandem mass spectrometry was performed to measure encapsulation efficiency of HPβCD C. Nanocarriers containing HPβCD (0.5 mM) were exposed on *Npc1*$^{-/-}$ for 24 hours, cells were fixed and filipin staining was performed to visualize reduction in lysosomal cholesterol.

Fig. S2 Effects of DSPE-PEG, HPBCD and their combination on cellular toxicity.

Npc1$^{-/-}$ MEFs cells were treated with DSPE-PEG_{2k} micelles (0-500 μM) and HPβCD (0-10 mM). Cells were fixed and stained with deep red nuclear mask. The cell number was calculated as a function of different treatment groups. The combinations were relatively non-toxic, a significant decrease in cell number was only observed at the highest combination concentration.

Fig. S3 DSPE-PEG shows enhanced cholesterol clearance with longer PEG chain lengths.

MEF *Npc1*$^{-/-}$ cells were treated for 24 hour with 10 μM of DSPE-PEG_{350}, DSPE-PEG_{1k}, DSPE-PEG_{2k}, DSPE-PEG_{5k}, DSPE-PEG_{10k}, DSPE-PEG_{20k}, DSPE-PEG_{30k} and DSPE or PEG_{2k} (unconjugated), 16 images per well (n=3) were collected and representative images are shown.

Fig. S4 Effects of Pluronic block copolymer on cholesterol clearance.

We exposed two concentrations (1.5 mg/ml and 0.15 mg/ml) of Pluronic block copolymers-L64, P84, P103 and F127 on *Npc1* deficient cells for 24 hours. Cells were fixed and stained for filipin and imaged using the EVOS microscope. (n=5 images were captured).

Fig. S1

A.

HSPC:DSPE-PEG$_{2k}$ Liposomes

95:5 50:50 25:75

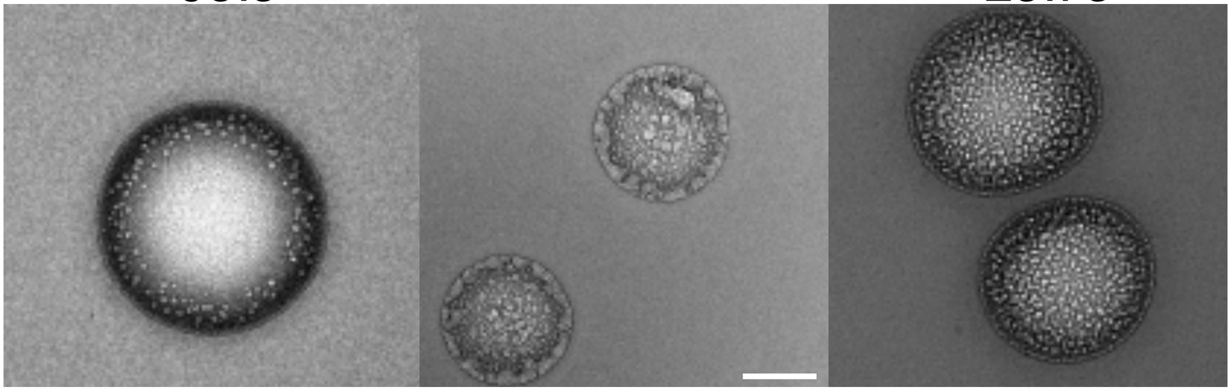

B.

Liposomal Composition	Average Size (DLS)	Loading Efficiency
95 % HSPC<br>5 % DSPE-PEG$_{2k}$	255 nm	22.9 %
50 % HSPC<br>50 % DSPE-PEG$_{2k}$	15.69 nm	35.7 %
25 % HSPC<br>75 % DSPE-PEG$_{2k}$	13.54 nm	30.9 %
100 % DSPE-PEG$_{2k}$	11.7 nm	10.4 %

C.

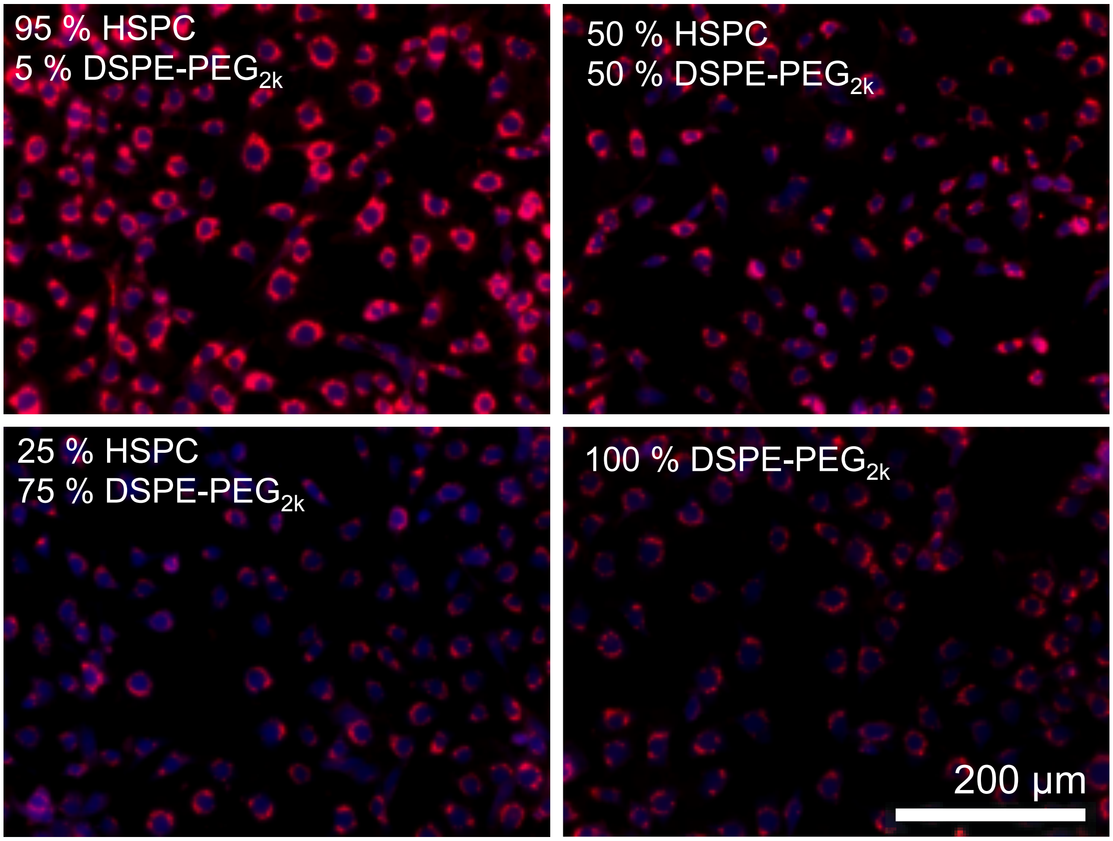

Fig. S2

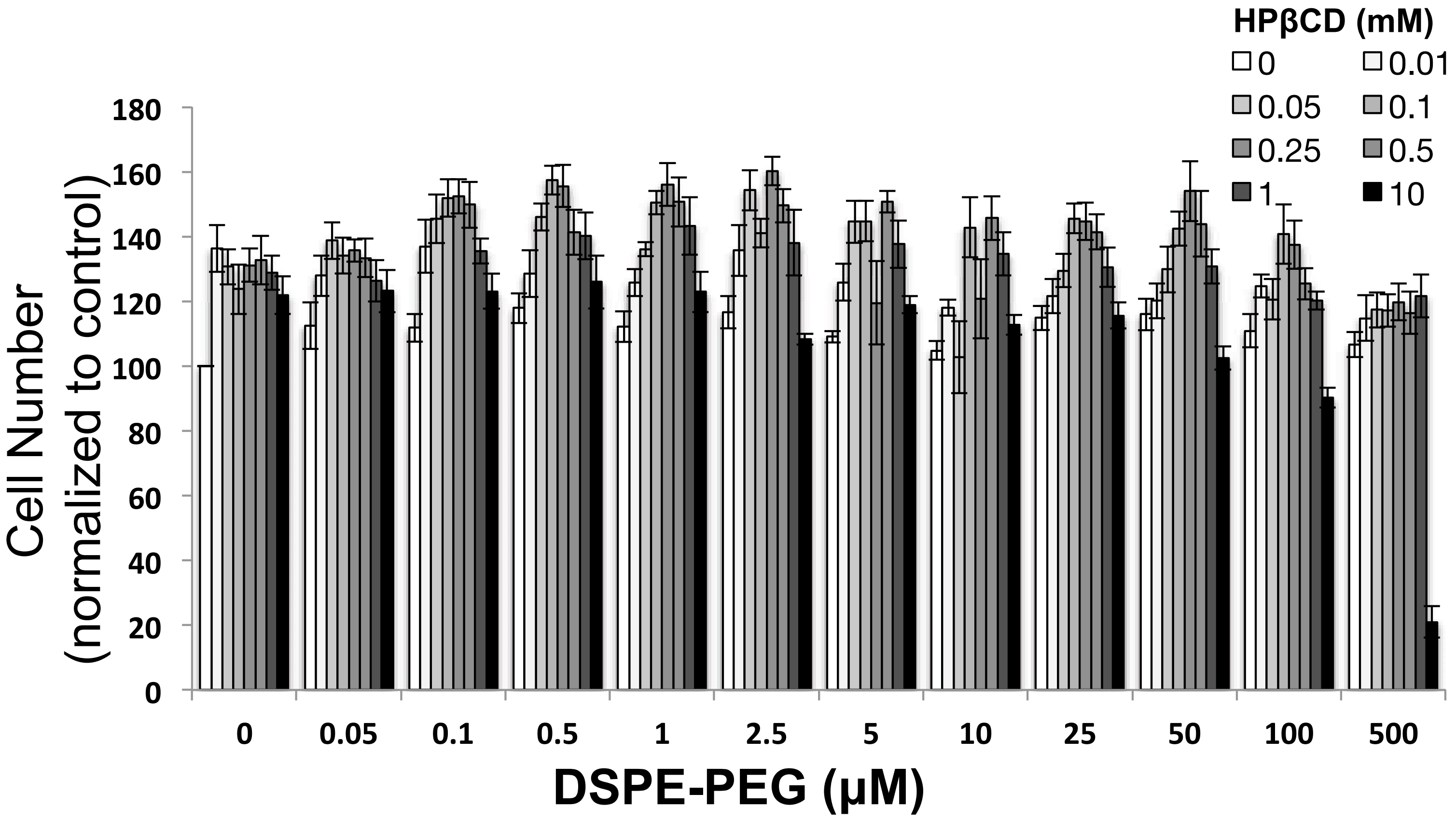

HPβCD (mM)
0
0.01
0.05
0.1
0.25
0.5
1
10
Cell Number (normalized to control)
180
160
140
120
100
80
60
40
20
0
0
0.05
0.1
0.5
1
2.5
5
10
25
50
100
500
DSPE-PEG (μM)

Fig. S3

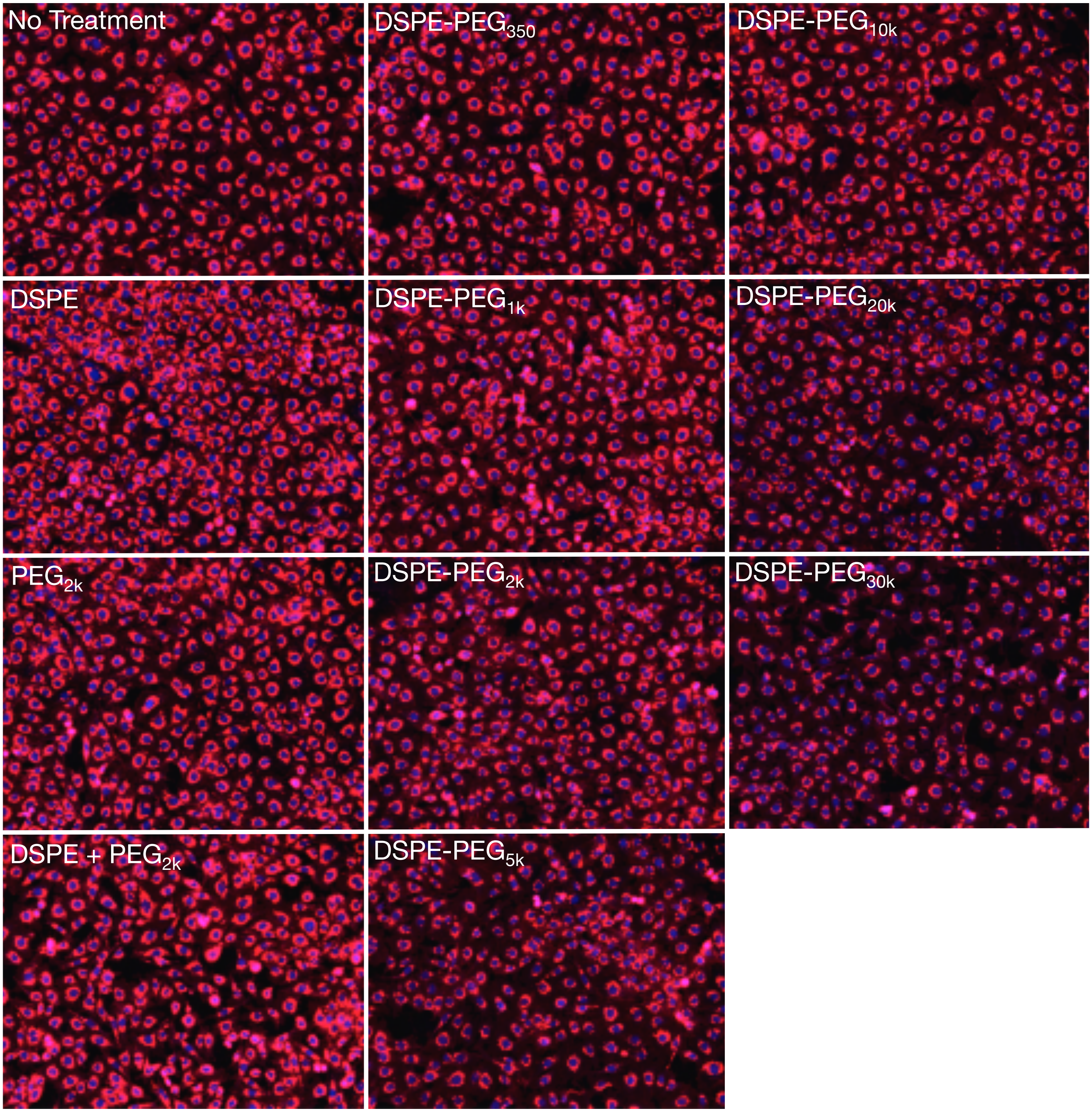
No Treatment
DSPE-PEG$_{350}$
DSPE-PEG$_{10k}$
DSPE
DSPE-PEG$_{1k}$
DSPE-PEG$_{20k}$
PEG$_{2k}$
DSPE-PEG$_{2k}$
DSPE-PEG$_{30k}$
DSPE + PEG$_{2k}$
DSPE-PEG$_{5k}$

Fig. S4

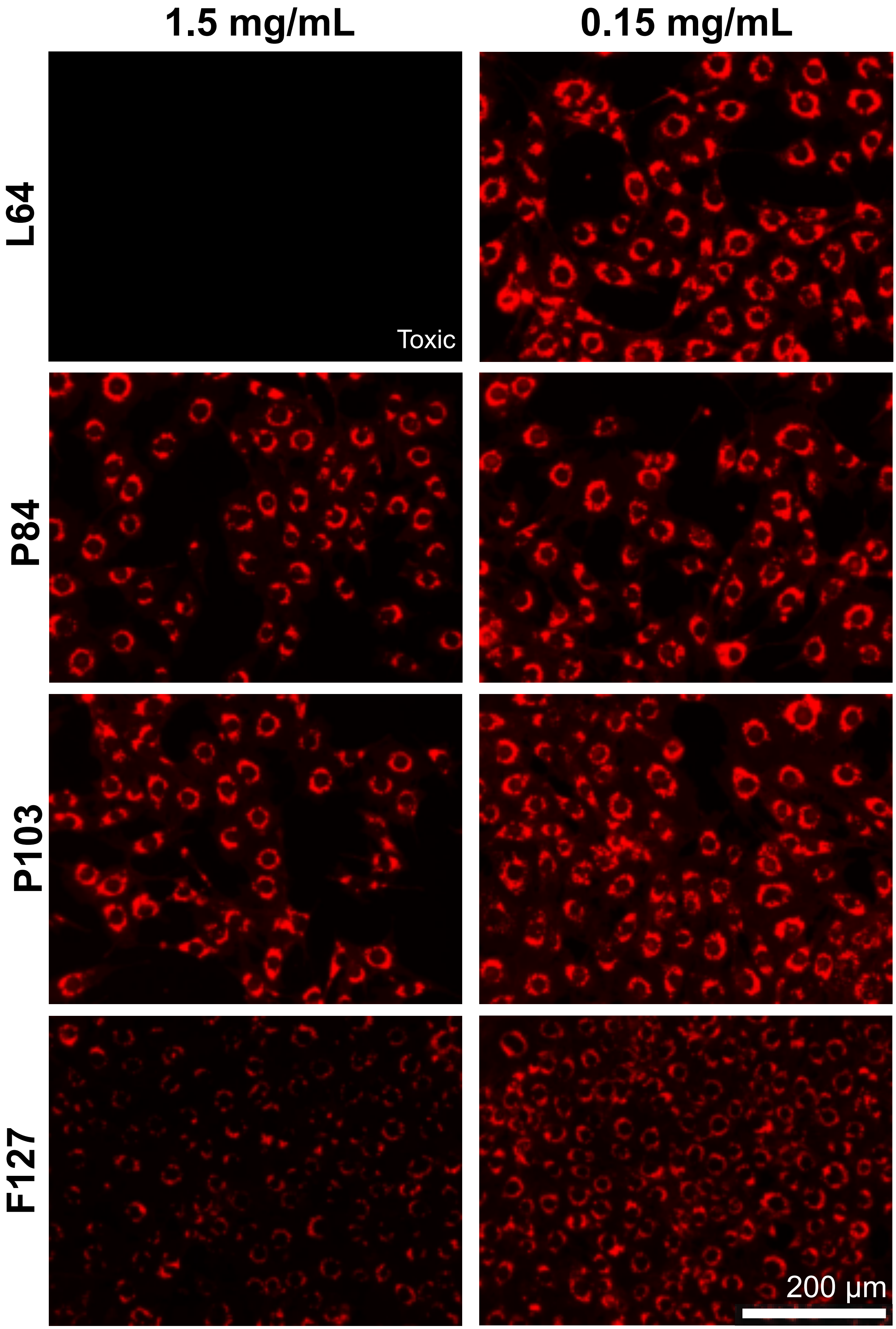

Supplement: Supplementary Information [file srep31750-s1.pdf]
